# Developing mental health curricula and a service provision model for clinical associates in South Africa: a Delphi survey of family physicians and psychiatrists

**DOI:** 10.1186/s12909-024-05637-2

**Published:** 2024-06-17

**Authors:** Saiendhra Vasudevan Moodley, Jacqueline Wolvaardt, Christoffel Grobler

**Affiliations:** School of Health Systems and Public Health, University of Pretoria, Pretoria, South Africa

**Keywords:** Mental health, Mental illness, Clinical associates, Task sharing, Delphi method, Delphi panel, Curriculum, Training

## Abstract

**Background:**

Clinical associates are a health professional cadre that could be utilised in mental health task sharing in South Africa but this is training dependent. The objectives of the study were to identify the potential curricula content, training sites, and teaching modalities for undergraduate and potential postgraduate clinical associate mental health training and to identify the tasks that they should perform based on these curricula.

**Methods:**

We utilised the Delphi method to reach consensus on items with the panel comprising psychiatrists and family physicians. The first round questionnaire of the Delphi survey was developed based on a literature review and the results from earlier phases of the overall study. The survey was administered electronically and consisted of three rounds. Following both the first and second rounds, an updated questionnaire was constructed omitting the items on which consensus was reached. The questionnaire consisted primarily of nine-point scales with consensus based on 70% of participants rating 1,2,3 or 7,8,9.

**Results:**

There were 26 participants in the first round with this number falling to 23 in later rounds. There was strong consensus on a training attachment to a mental health clinic at a community health centre (CHC) at undergraduate (96.2%) and postgraduate level (100%). Consensus was reached on the importance of training on the management of six categories of disorders at the undergraduate level and nine categories of disorders at the postgraduate level. Clerking patients as a teaching modality reached 100% consensus at both undergraduate and postgraduate levels. PHC clinics, CHCs and district hospitals reached consensus as appropriate settings for clinical associates to provide mental health services. In addition, GP practices and secondary hospitals reached consensus for those with postgraduate training. Consensus was reached on ten of the 21 listed tasks that could be performed based on undergraduate training and 20 of the 21 tasks based on a postgraduate qualification in mental health.

**Conclusions:**

The Delphi panel’s recommendations provide a clear roadmap for enhancing mental health curricula for clinical associates, enabling their utilisation in mental health service provision. A future postgraduate mental health qualification for clinical associates would allow for expanded task sharing.

## Background

South Africa’s health system comprises public and private sectors with service delivery in the public sector largely being the responsibility of the nine provincial government health departments [1]. The under-resourced public sector serves more than 80% of the population while the private sector caters for a minority, mainly-insured, population [2–4]. Disparities in access to health services exist between the public and private sectors, between urban and rural areas, and between provinces [4]. These disparities are reflected in access to mental health services with the majority of psychiatrists, for example, based in the private sector and in urban areas [5]. Psychiatrists and psychologists in the private sector can be accessed by the insured population and those who can afford out-of-pocket payments. In contrast, there are system-wide shortages of specialist mental health professionals in the public sector [6]. Mental health services in the public sector in South Africa should be provided at all levels of care [7]. The resources are, however, heavily skewed to higher levels of care [4, 6]. Specialised psychiatric hospitals accounted for 45.0% of total public sector mental healthcare expenditure in 2016/17 compared to 11.7% of total public sector mental healthcare expenditure at the district hospital level and only 7.9% of total public sector mental healthcare expenditure for outpatient services at the primary care level [6].

Given the critical shortage of specialist mental health professionals in the public sector in South Africa, a need for mental health task sharing models has been identified [4]. Clinical associates, the mid-level medical worker cadre in South Africa, are a potential option in mental health task sharing. This task sharing could help address access to mental health services given the high prevalence of mental illness in South Africa [8, 9] by supplementing the current mental health workforce and addressing the inequitable distribution of the workforce [5, 6, 10–14]. The development of the clinical associate cadre is relatively recent in South Africa with the first graduates entering the South African health system in 2011 [15]. The cadre was created primarily to address the health workforce shortages at the district level which result in a lack of access to health services particularly in the rural parts of South Africa [15]. The scope of practice of clinical associates include history taking, conducting physical examinations, ordering or performing certain investigations, diagnosing and managing common conditions, and performing several procedures [16, 17]. Clinical associates are expected to work under the supervision of a medical doctor, with a degree of supervision commensurate with the clinical associates level of experience [15, 16]. The clinical associate profession faces several challenges in South Africa currently, including a lack of posts in the public sector, an absence of career pathing, and limited prescribing rights [17, 18]. Clinical associate undergraduate degrees are offered at three universities in South Africa with the degree named the Bachelor of Clinical Medical Practice (BCMP) at two universities and the Bachelor of Medicine in Clinical Practice (BMCP) at one university [17, 19]. All three offerings are three-year programmes and follow a broadly similar approach of early exposure to clinical settings and problem-based learning with district hospitals as the main training sites [15, 17]. The three universities jointly administer a national exit examination for final-year students [17].

Clinical associates’ undergraduate training in mental health determines the extent to which they could be used in mental health service provision. The mental health training within the three undergraduate clinical associate programmes has been reviewed by Moodley et al. [19]. Almost all of the mental health training in the three programmes takes place in the third year of the degree, but the allocated time and experiential learning differs between the programmes [19]. The time spent on formal lectures ranges from a few hours to a week and placement at a health facility ranges from two to four weeks [19]. All three programmes attempt to teach a long list of disorders, even those that are uncommon and are unlikely to be seen or managed by clinical associates [19]. The amount of practical exposure differs between programmes with one programme placing their students in a dedicated mental health unit thus ensuring adequate clinical exposure, limited practical exposure in the second programme, and considerable variation in the third programme depending on the site where students are placed [19]. Moodley et al. [19] recommended that mental health training in the undergraduate programmes could be improved by earlier integration in the curricula, focusing on common conditions, rotations to mental health units in all three programmes, providing detailed guidance to facility-based trainers, and including specific experiential targets for the students [19].

Although there is evidence of mid-level medical workers being involved in mental health service provision in Africa [20–22], it is not clear how much training these cadres receive in mental health during their training programmes. In the United States of America (USA), there is substantial variation in mental health training between the different physician assistant programmes [23]. The mental health training that Canadian physician assistants undergo is also not well documented, but based on their competency profile, they are expected to be able to recognise, diagnose, and treat anxiety, depression, eating disorders, and adjustment reactions (e.g. grief) as well as conduct a suicide assessment [24]. The physician associate programmes in the United Kingdom (UK) are only required to include a minimum of 90 h of psychiatry [25], which includes problem-based learning sessions and mental health consultations as part of general practice clinical placements [26].

It has been suggested that advanced training or specialisation in mental health through a postgraduate qualification (such as an Honours degree or diploma) should be a serious consideration for clinical associates as it would increase their utility in mental health service delivery [27]. Specialised clinical associates could help address the inequitable access to specialist mental health professionals and mental health services in rural areas while addressing the lack of career pathing, employment opportunities, and options for postgraduate study that are a cause of frustration among clinical associates [27]. Advanced training would also give clinical associates with a specific interest in mental health an opportunity to pursue their interest in the discipline [27]. A survey of clinical associates in South Africa found considerable interest in a specialisation in mental health [28]. Currently, the only postgraduate opportunity that exists specifically for clinical associates in South Africa is an Honours degree in emergency medicine [29]. A specialisation in mental health for similar cadres already exists in some African countries [22, 30, 31]. In Malawi, their mid-level medical worker cadre (clinical officers) completes a three-year diploma and one-year internship and can, thereafter, pursue advanced training in mental health through a two-year Bachelor of Science in Clinical Medicine (Mental Health) degree [22, 32]. They are then able to practise as specialised psychiatric clinical officers and form part of district mental health teams based at district hospitals [22]. A specialisation in psychiatry is possible in the USA through a one-year residency or fellowship and/or obtaining a Certificate of Added Qualification (CAQ) in psychiatry [23]. Psychiatry is one of seven specialties in the USA in which a physician assistant can obtain a CAQ [33]. Speciality is achieved through experience, continuing professional development, physician attestation as well as the successful completion of a national exam [33].

Clinical associates in South Africa are already involved, to some extent, in mental health service provision [34]. While they have good self-reported knowledge, there is a lack of confidence concerning certain aspects of practical application in assessing and managing mental health patients [34], and undergraduate training needs to address some of the deficiencies for future graduates [19, 34]. In addition, the rollout of a postgraduate qualification in mental health for clinical associates needs to be considered to address shortages in the specialist mental health workforce and provide career progression opportunities for clinical associates [27]. The objectives of the study were to identify the potential curricula content, training sites, and teaching modalities for undergraduate and postgraduate clinical associate mental health training in South Africa and to identify the mental health tasks that clinical associates should perform based on these curricula.

## Methods

### Study design

We utilised the Delphi method in the third phase of a larger study to reach consensus on training items and mental health tasks to include in a mental health task-sharing model for clinical associates.

### Study population and sampling

The study population consisted of psychiatrists and family physicians working either in the public or private sector within the South African health system. Purposive sampling was utilised to select the Delphi participants. Psychiatrists and family physicians were selected to ensure differing levels of experience and participants were invited from different provinces. The target was to include a minimum of 10 psychiatrists and 10 family physicians in the panel.

### Measurement tools

The items for Round 1 of the Delphi survey were developed based on a literature review as well as the results from the first two phases of the larger study [19, 27, 34, 35]. The first round of questionnaires included the professional characteristics of the members of the Delphi panel. The training components of the questionnaire comprised the levels of care at which mental health training of clinical associates should occur, the mental illnesses they should be able to recognise and manage based on the Diagnostic and Statistical Manual of Mental Disorders, Fifth Edition (DSM-5) [36] categories, and the teaching modalities and health professionals that should be utilised in training. Service provision comprised the levels of care at which mental health task sharing should occur, and the mental health tasks that could be performed. The questions were asked for both undergraduate and postgraduate training. Participants were asked to assume that only four weeks (160 notional hours) could be allocated to mental health in the BCMP/BMCP curriculum for the questions related to undergraduate training. Participants were asked to consider a potential postgraduate mental health training qualification (one-year full-time or two-year part-time postgraduate diploma or Honours degree) for the questions related to postgraduate training. The questionnaire consisted primarily of nine-point scales (1–9). Depending on the question, these ranged from extremely unimportant to extremely important, extremely unsuitable to extremely suitable, or extremely inappropriate to extremely appropriate. In the first round, participants were also requested to suggest additional items to add to the questionnaire. Items suggested by two or more participants were included in the second round questionnaire.

### Data collection

The College of Psychiatrists and the College of Family Physicians of the Colleges of Medicine of South Africa as well as the Heads of Departments of Family Medicine at South Africa’s medical schools, assisted identifying potential panellists. Potential participants were contacted by e-mail and requested to participate. The Delphi survey was administered electronically using the Qualtrics platform and it was decided before commencement that it would consist of a maximum of three rounds, with the survey only being terminated after the first or second round if there was consensus on all items. It was decided not to go beyond three rounds, as Iqbal and Pipon-Young [37] have noted that multiple rounds impact enthusiasm and response rates. Participants were provided with links to the scope of practice of clinical associates [16], a paper by Moodley et al. [19] on mental health in the current clinical associate undergraduate curriculum, and a list of disorders making up each DSM-5 [36] category as background information. A video recording of what was required from participants was provided to them prior to Round 1 and a new recording was provided before Round 2. They were given approximately two weeks to submit their responses in each round with periodic reminders of upcoming deadlines. An updated questionnaire was constructed and sent out to the panellists to complete after the first round as well as the second round. Questions on which consensus was reached were not included in subsequent rounds. Questionnaires in the second and third rounds included the participant’s response for each item from the previous round as well as the median response of the panel for each item. The participants were, therefore, reminded of their response compared to the rest of the panel before revisiting the question [38, 39].

### Data management and analysis

The data were downloaded from Qualtrics after each round and stored on a password-protected computer with a backup stored in a password-secured cloud account. The in-built Qualtrics functionality was used to determine the proportions of each response (1–9) on the scales being used. Consensus was based on a proportion within a range (unrestricted) approach, with 70% of participants rating 1,2,3 or 7,8,9 regarded as consensus [40]. Data were imported into Stata version 16 (Statacorp; http://www.stata.com) after the first two rounds to determine the median panel responses for each item to include in the questionnaire in the subsequent round.

### Processes to ensure quality of research

Follow-up e-mails were sent to participants to ensure non-responses in each round were kept to a minimum [41]. The four criteria of a good quality Delphi study suggested by Diamond et al. [40] were adhered to. The planned number of survey rounds was specified, stopping criteria were specified, reproducible criteria were used for the selection of participants, and the criteria to be used to drop items after each round were provided [40].

## Results

The first round of the Delphi survey involved 26 participants (15 psychiatrists and 11 family physicians). These 26 participants were invited to participate in the second round, but this was completed by only 23 of them (13 psychiatrists and 10 family physicians). Only the 23 participants who completed the second round were invited to participate in the third round, with all of them completing the third round. The dropout rate was, therefore, 11.5% (3/26). The professional characteristics of the participants are shown in Table 1. Five of South Africa’s nine provinces were represented, with the largest number of participants from Gauteng.

**Table 1:** Professional characteristics of the Delphi participants

	Round 1 *N* = 26 *n* (%)	Rounds 2 & 3 *N* = 23 *n* (%)
**Occupational category**		
Family Physician	11 (42.3)	10 (43.5)
Psychiatrist	15 (57.7)	13 (56.5)
**Years of experience as a specialist**		
0–9 years	7 (26.9)	7 (30.4)
10–19 years	12 (46.2)	10 (43.5)
20–29 years	4 (15.4)	3 (13.0)
30 years or more	3 (11.5)	3 (13.0)
**Sector of work**		
Public sector only	17 (65.4)	15 (65.2)
Private sector only	3 (11.5)	3 (13.0)
Both public and private sectors	5 (19.2)	4 (17.4)
Other	1 (3.8)	1 (4.3)
**Province of work**		
Eastern Cape	1 (3.8)	1 (4.3)
Gauteng	12 (46.2)	11 (47.8)
Kwazulu Natal	5 (19.2)	3 (13.0)
Limpopo	2 (7.7)	2 (8.7)
Western Cape	6 (23.1)	6 (26.1)

### Training

#### Training attachments

Participants were asked to rate the importance of various training attachments in an undergraduate BCMP/BMCP curriculum as well as a postgraduate mental health qualification for clinical associates (Table 2). A nine-point scale was used, i.e. extremely unimportant (1) to extremely important (9). The only item that did not reach consensus in the first round for undergraduate training was attachment to an inpatient unit at a psychiatric hospital. This item also failed to reach consensus in the second and third rounds. A mental health attachment to a primary health care (PHC) clinic for undergraduate training was added to the second round following feedback from the participants in the first round and this item reached consensus in the second round. The only item that did not reach consensus for postgraduate training was attachment to a private psychiatric practice (Table 2).

**Table 2:** Importance of potential mental health training attachments at undergraduate and postgraduate levels for clinical associates (all items)

Attachment	Proportion of participants scoring 7,8,9 (%)		
	Round 1	Round 2	Round 3
**Potential training attachments in an undergraduate BCMP/BMCP curriculum**	***N*** ** = 26**	***N*** ** = 23**	***N*** ** = 23**
Attachment to a mental health clinic at a community health centre	96.2	-	-
Attachment to a psychiatric outpatients clinic at a hospital	84.6	-	-
Attachment to a 72-hour observation unit at a district hospital	73.1	-	-
Attachment to inpatient unit at a psychiatric hospital	30.8	30.4	4.3
Mental health attachment to a primary health care clinic	-	82.6	-
**Potential training attachments in a postgraduate mental health qualification for clinical associates**	***N*** ** = 25**	***N*** ** = 23**	***N*** ** = 23**
Attachment to a mental health clinic at a community health centre	100.0	-	-
Attachment to a psychiatric outpatients clinic at a hospital	100.0	-	-
Attachment to a 72-hour observation unit at a district hospital	100.0	-	-
Attachment to inpatient unit at a psychiatric hospital	72.0	-	-
Attachment to a private psychiatric practice	28.0	8.7	8.7

##### Training on the recognition and management of psychiatric disorders

The participants rated the importance of training on the recognition and management of the 19 DSM-5 [36] categories of psychiatric disorders as part of undergraduate BCMP/BMCP curricula. Consensus was reached in the first round regarding the importance of recognising eight categories of disorders at the undergraduate level (Table 3). Neurocognitive disorders reached consensus in the second round, with no further categories reaching consensus in the third round. Consensus was reached on the importance of training (rating 7,8 or 9) to manage depressive disorders, anxiety disorders, substance-related and addictive disorders in the first round and a further three categories of disorders in the second round (Table 3). The management of paraphilic disorders (82.6%) and dissociative disorders (73.9%) reached consensus in being considered unimportant (rating 1, 2 or 3) in the third round .

Similarly, the 19 DSM-5 [36] categories were provided to participants for a hypothetical postgraduate mental health qualification for clinical associates. In the first round of the Delphi, nine categories reached consensus (rating 7, 8 or 9) regarding training to recognise disorders with an additional three categories reaching consensus in the third round of the survey (Table 3). Consensus was reached on the importance of training (rating 7,8 or 9) regarding the management of eight categories of disorders in the first round and neurocognitive disorders in the second round with no additional categories of disorders reaching consensus in the third round (Table 3).

**Table 3:** Importance for training on the recognition and management of psychiatric disorders at undergraduate and postgraduate levels (items where consensus on being important was reached)

DSM-5 disorder categories	Proportion of participants scoring 7,8,9 (%)		
	Round 1	Round 2	Round 3
**Training on recognition in an undergraduate BCMP/BMCP curriculum**	***N*** ** = 26**	***N*** ** = 23**	***N*** ** = 23**
Depressive disorders	100.0	-	-
Bipolar and related disorders	100.0	-	-
Schizophrenia spectrum and other psychotic disorders	100.0	-	-
Substance-related and addictive disorders	96.2	-	-
Anxiety disorders	96.0*	-	-
Trauma- and stressor-related disorders	92.3	-	-
Neurodevelopmental disorders	80.8	-	-
Medication-induced movement disorders and other adverse effects of medication	76.9	-	-
Neurocognitive disorders	65.4	78.3	-
**Training on management in an undergraduate BCMP/BMCP curriculum**	***N*** ** = 26**	***N*** ** = 23**	***N*** ** = 23**
Depressive disorders	88.4	-	-
Anxiety disorders	84.6	-	-
Substance-related and addictive disorders	80.8	-	-
Trauma and stressor related disorders	65.4	78.3	-
Bipolar and related disorders	61.5	78.3	-
Schizophrenia spectrum and other psychotic disorders	60.0*	78.3	-
**Training on recognition in a postgraduate mental health qualification for clinical associates**	***N*** ** = 25**	***N*** ** = 23**	***N*** ** = 23**
Depressive disorders	100.0	-	-
Substance-related and addictive disorders	100.0	-	-
Anxiety disorders	100.0	-	-
Trauma and stressor related disorders	100.0	-	-
Bipolar and related disorders	92.0	-	-
Schizophrenia spectrum and other psychotic disorders	92.0	-	-
Medication-induced movement disorders and other adverse effects of medication	84.0	-	-
Neurodevelopmental disorders	80.0	-	-
Neurocognitive disorders	80.0	-	-
Sleep-wake disorders	64.0	69.6	78.3
Somatic symptom and related disorders	60.0	60.9	73.9
Obsessive-compulsive and related disorders	60.0	56.5	73.9
**Training on management in a postgraduate mental health qualification for clinical associates**	***N*** ** = 25**	***N*** ** = 23**	***N*** ** = 23**
Depressive disorders	92.0	-	-
Anxiety disorders	92.0	-	-
Substance-related and addictive disorders	87.5^#^	-	-
Trauma and stressor related disorders	84.0	-	-
Bipolar and related disorders	79.2^#^	-	-
Schizophrenia spectrum and other psychotic disorders	76.0	-	-
Medication-induced movement disorders and other adverse effects of medication	76.0	-	-
Neurodevelopmental disorders	72.0	-	-
Neurocognitive disorders	68.0	78.3	-

*only answered by 25 participants (*N* = 25), ^#^only answered by 24 participants (*N* = 24)

##### Teaching modalities

Potential teaching modalities for mental health were provided to the participants to rate their suitability for mental health training in the undergraduate BCMP/BMCP curriculum as well as postgraduate mental health qualification. Consensus was reached in the first round for suitability (7,8,9) for all potential teaching modalities at the undergraduate level except attending patient rounds, watching recording consultations, and online lectures, which were all just under 70.0% (Table 4). These three items reached consensus in the second round. All potential teaching modalities for a postgraduate mental health qualification for clinical associates reached consensus in the first round. (Table 4).

**Table 4:** Suitability of potential teaching modalities at undergraduate and postgraduate levels for clinical associates (all items)

Teaching modalities	Proportion of participants scoring 7,8,9 (%)		
	Round 1	Round 2	Round 3
**Potential teaching modalities in an undergraduate BCMP/BMCP curriculum**	***N*** ** = 26**	***N*** ** = 23**	***N*** ** = 23**
Clerking patients	100.0	-	-
Case vignettes to formulate differential diagnoses	96.2	-	-
Case studies	92.3	-	-
Simulated mental health assessments	88.5	-	-
Attending multidisciplinary team meetings	80.8	-	-
In-person lectures	76.9	-	-
Attending patient rounds	69.2	78.3	-
Watching recording consultations	69.2	73.9	-
Online lectures	69.2	73.9	-
Sitting in on consultations	-	87.0	-
**Potential teaching modalities in a postgraduate mental health qualification for clinical associates**	***N*** ** = 25**	***N*** ** = 23**	***N*** ** = 23**
Clerking patients	100.0	-	-
Case studies	100.0	-	-
Attending multidisciplinary team meetings	100.0	-	-
Attending patient rounds	100.0	-	-
Case vignettes to formulate differential diagnoses	96.0	-	-
Simulated mental health assessments	96.0	-	-
In-person lectures	91.3*	-	-
Watching recorded consultations	80.0	-	-
Online lectures	76.0	-	-

*Only answered by 23 participants (*N* = 23)

##### Health professionals involved in training

The participants were asked to rate the importance of involving various health professional cadres in the mental health training of clinical associates in undergraduate BCMP/BMCP programmes. Consensus on importance (7,8, or 9) was reached in the first round (*N* = 26) for family physicians (84.6%), psychologists (84.6%), qualified clinical associates (76.9%), psychiatrists (73.1%), and psychiatric nurses (73.1%). The three cadres that did not reach consensus in the first round were psychiatric registrars (69.2%), family medicine registrars (65.4%), and medical officers (61.5%). Consensus was reached on psychiatric registrars (87.0%) and family medicine registrars (87.0%) in the second round (*N* = 23) and on medical officers (82.6%) in the third round (*N* = 23). Following participant feedback, social workers (60.9%) and occupational therapists (52.2%) were added as items in the second round. For both social workers (87.0%) and occupational therapists (73.9%), consensus was only reached in the third round.

Consensus on the importance (7,8,9) of involving various health professionals cadres in the training of clinical associates in postgraduate mental health qualification was reached in the first round (*N* = 25) for family physicians (88.0%), psychiatrists (88.0%), psychologists (80.0%), psychiatric registrars (80.0%), and psychiatric nurses (76.0%). No consensus was reached on family medicine registrars (68.0%) and medical officers (44.0%) in the first round. Social workers and occupational therapists were also added to the second round of postgraduate training following participant feedback. Only family medicine registrars (87.0%) reached consensus in the second round (*N* = 23) and social workers (78.3%) in the third round (*N* = 23) of the remaining cadres. No consensus was reached on medical officers and occupational therapists.

### Service provision

#### Work settings for service provision

The Delphi panel reached consensus in the first round (Table 5) on CHCs, PHC clinics, and district hospitals as appropriate settings (7,8,9) for clinical associates to deliver mental health services based on undergraduate training. No consensus was reached on any of the other work settings in the subsequent rounds. Specialised psychiatric hospitals (69.6%) fell just short of the 70.0% consensus threshold for being considered inappropriate (1,2,3). The Delphi panel reached consensus in the first round on CHCs, PHC clinics, district hospitals, and private GP practices as appropriate settings (7,8,9) for clinical associates to deliver mental health services based on postgraduate training. (Table 5). Consensus was reached on secondary hospitals in the second round, with no additional work setting reaching consensus in the third round.

**Table 5:** Appropriateness of potential work settings for clinical associates to provide mental health services (all items)

Work setting	Proportion of participants scoring 7,8,9 (%)		
	Round 1	Round 2	Round 3
**With only undergraduate mental health training as part of BCMP/BMCP degrees**	***N*** ** = 26**	***N*** ** = 23**	***N*** ** = 23**
Community health centre	92.3	-	-
Primary health care clinic	88.5	-	-
District hospital	76.9	-	-
GP Practice	46.2	34.8	13.0
Secondary hospital	38.5	30.4	8.7
Psychiatrist practice	19.2	0.0	0.0
Tertiary hospital	15.4	0.0	0.0
Private hospital	15.4	0.0	0.0
Specialist psychiatric hospital	11.5	0.0	0.0
**With a postgraduate qualification in mental health**	***N*** ** = 25**	***N*** ** = 23**	***N*** ** = 23**
Community health centre	96.0	-	-
District hospital	92.0	-	-
Primary health care clinic	88.0	-	-
GP Practice	76.0	-	-
Secondary hospital	68.0	78.3	-
Specialist psychiatric hospital	32.0	17.4	0.0
Psychiatrist practice	32.0	4.4	0.0
Tertiary hospital	28.0	17.4	4.4
Private hospital	24.0	13.6	4.4

##### Mental health tasks

The Delphi panel reached consensus on eight of the 21 listed mental health tasks for clinical associates with undergraduate degrees in the first round and one additional task in Round 2 (Table 6). The same 21 mental health tasks were provided to the panel to rate the appropriateness for clinical associates with a postgraduate mental health qualification to perform. A total of 16 tasks reached consensus for appropriateness in the first round, one task in the second round, and three tasks in the third round (Table 6). The only task that did not reach consensus was completing the required reports for patients admitted to the 72-hour observation units (69.6%) which was marginally below the consensus threshold of 70.0%.

**Table 6:** Appropriateness of mental health tasks that could potentially be performed by clinical associates

Work setting	Proportion of participants scoring 7,8,9 (%)		
	Round 1	Round 2	Round 3
**With only undergraduate mental health training as part of BCMP/BMCP degrees (items where consensus on being appropriate was reached)**	***N*** ** = 26**	***N*** ** = 23**	***N*** ** = 23**
Screening for common mental disorders	96.2	-	-
Taking a mental health history from patients suspected of having a mental illness	96.2	-	-
Doing a mental health examination on patients suspected of having a mental illness	92.3	-	-
Mental health promotion in communities	88.5		
Mental health promotion in schools	84.6	-	-
Doing physical examination on patients with mental illness	80.8	-	-
Assessing cognitive functioning using a suitable cognitive screening test on patients suspected of having a mental illness	80.8	-	-
Home visits to mental health patients	76.9	-	-
Providing counselling to families of patients with mental illness	-	86.9	-
Providing counselling to patients with mental illness	**-**	78.3	**-**
**With a postgraduate qualification in mental health (all items)**	***N*** ** = 25**	***N*** ** = 23**	***N*** ** = 23**
Taking a mental health history from patients suspected of having a mental illness	100.0	-	-
Doing a mental health examination on patients suspected of having a mental illness	100.0	-	-
Screening for common mental disorders	96.0	-	-
Assessing cognitive functioning using a suitable cognitive screening test on patients suspected of having a mental illness	96.0	-	-
Providing counselling to families of patients with mental illness	96.0	-	-
Mental health promotion in communities	96.0	-	-
Mental health promotion in schools	96.0	-	-
Providing counselling to patients with mental illness	95.8*	-	-
Doing physical examination on patients with mental illness	88.0	-	-
Home visits to mental health patients	84.0	-	-
Monitoring of patients admitted to 72-hour observation units	76.0	-	-
Management of a patient suspected to be exposed to traumatic event(s)	75.0*	-	-
Management of a patient at risk of suicide	73.9^#^	-	-
Managing common side effects from psychiatric medication	72.0	-	-
Management of a patient presenting with confusion	70.8*	-	-
Management of a patient presenting with aggression	70.8*	-	-
Restraining a patient who is aggressive/violent	68.0	73.9	-
Sedating a patient who is aggressive/violent	64.0	69.6	82.6
Prescribing psychotropic medication to patients with mental illness	56.0	65.2	73.9
Managing serious adverse events from emergency psychiatric medication	52.0	56.5	73.9
Completing the required reports for patients admitted to 72-hour observation units	66.7*	65.2	69.6

*Only answered by 24 participants (*N* = 24), ^#^only answered by 23 participants (*N* = 23)

## Discussion

Our study aimed to identify the potential curricula content, training sites, and teaching modalities for undergraduate and postgraduate training in mental health for clinical associates and identify the mental health tasks that clinical associates should perform based on these curricula. The identified items would form part of a mental health task sharing model for clinical associates. Mental health training currently forms part of the three BCMP/BMCP curricula in the country [19] but there is no existing postgraduate qualification in mental health for clinical associates. The panel was, therefore, asked to consider a hypothetical postgraduate qualification (postgraduate diploma or Honours degree). Unsurprisingly, the panel drew a clear distinction between the content that should be included at an undergraduate level compared to a postgraduate level as well as the tasks that should be performed by those with only undergraduate training in mental health compared to those with a postgraduate qualification.

There was strong consensus from the panel on the importance of attachments to a mental health clinic at a CHC for both undergraduate and postgraduate training. In general, clinical associate training across all disciplines takes place at the hospital level [15, 17] and PHC attachments would require an overall shift in approach. There is no evidence that CHCs are currently being utilised for undergraduate mental health training, instead the most common sites for their mental health rotation are district and regional hospitals [34]. South Africa’s National Mental Health Policy Framework and Strategic Plan 2023–2030 [42] indicates that community mental health services, including mental health services at primary health care facilities, will be scaled up. This strategy presents an opportunity to make use of CHCs and PHC clinics that provide mental health services for clinical associate training. The benefits of mental health attachments to PHC settings may include exposure to a broader range of patients, developing an approach that is more ‘patient-centred’, and reducing stigmatising attitudes [43]. Primary care settings are used in the USA and UK for the mental health training of physician assistants and physician associates, respectively [23, 26]. The process of developing PHC attachments for clinical associates in mental health and other disciplines would require similar processes to those followed in establishing district hospital sites including negotiations with the relevant provincial authorities, identifying suitable sites, and developing preceptors for the students [15].

The panel was provided with the 19 DSM-5 [36] categories for undergraduate curriculum content. The six disorder categories reaching consensus (for clinical associates playing a role in management) appear to be linked to their prevalence or burden of disease. Based on the global burden of disease data for 2019, depressive disorders ranked 13th and were the highest ranked of the mental disorders with respect to the leading causes of disability-adjusted life-years (DALYs) followed by anxiety disorders (ranked 24th ), and schizophrenia (ranked 42nd ) [44]. Depressive disorders (ranked 2nd ), anxiety disorders (ranked 8th ), and schizophrenia (ranked 20th ) were also the leading mental health disorders when considering years lived with disability (YLDs) [44]. Bipolar disorder also featured prominently in the DALYs (ranked 67th ) and YLDs (ranked 28th ) [44]. Herman et al. [8] reported that the three mental disorders with the highest lifetime prevalence in South Africa were anxiety disorders (15.8%), substance use disorders (13.3%), and mood disorders (9.8%). The consensus reached on trauma- and stressor-related disorders is also not surprising given the high levels of interpersonal violence, including gender-based violence, in South Africa [45]. The items on which the panel reached consensus support previous recommendations by Moodley et al. [19] that questioned the utility of including several uncommon disorders in the clinical associate undergraduate mental health curriculum and recommended a focus on common and high-burden disorders. The scope of practice of clinical associates in South Africa does not specify the mental health disorders that the cadre is expected to recognise and manage [16]. Physician assistants in Canada are expected to recognise and manage anxiety, depression, adjustment reactions, and eating disorders [24]. Feeding and eating disorders did not reach consensus among our panel for recognition or management suggesting the panel perceived these disorders to be low priority in South Africa. In a survey conducted among clinical associates in South Africa, self-reported knowledge deficits were identified for schizophrenia, dementia, acute stress disorder, bipolar disorders and attention-deficit hyperactivity disorder, which all fall within the DSM-5 categories on which consensus was reached regarding recognition, suggesting that undergraduate training on these disorder needs to strengthened [34].

Three additional disorder categories (medication-induced movement disorders and other adverse effects of medication, neurodevelopmental disorders, and neurocognitive disorders) were considered important for training on management at the postgraduate level compared to the undergraduate level. Sorsdahl et al. [4] have noted the neglect and inequitable distribution of child and adolescent mental health as well as psychogeriatric services in South Africa and the consensus reached by the panel on training on the management of neurodevelopmental disorders and neurocognitive disorders suggests clinical associates with a postgraduate qualification in mental health may have a role to play in addressing this neglect. While clinical associates with only undergraduate training in mental health might not be expected to manage neurodevelopmental and neurocognitive disorders, the panel did reach consensus on the importance of recognising these conditions. A recently developed competence framework for physician associates working in mental health in the United Kingdom emphasises the importance of being able to recognise presentations across the lifespan, including knowledge of a range of neurodevelopmental and neurodegenerative conditions [46, 47].

All potential teaching modalities provided to the panel reached consensus for both undergraduate and postgraduate training. The only item that reached 100% consensus at an undergraduate level was clerking patients highlighting the importance of practical training. Practical learning is mainly by chance in two of the three BCMP/BMCP programmes in the country, and this would need to be addressed [19]. It has been found in a study among medical students that compared several different teaching modalities during their psychiatry rotation that face-to-face clinical teaching with psychiatrists was the most highly valued by the students [48]. While the COVID-19 pandemic has seen the wider use of online teaching in psychiatry for medical students, for example [49, 50], online lectures had the lowest proportion of consensus amongst the panel for suitability at both undergraduate and postgraduate levels, though it did reach the 70% consensus threshold. It has been suggested by Nayak [51] that using “newer” teaching techniques in psychiatry such as case-based and problem-based learning, simulated patients, multidisciplinary seminars, movies, and small group teaching could develop interest in psychiatry among medical students and reduce stigmatising attitudes. Problem-based learning, which is extensively utilised in clinical associate training [17], is an effective approach in undergraduate medical education generally [52] and has been found to significantly improve examination performance in psychiatry among medical students [53].

Currently, the mental health training of undergraduate clinical associates at health facilities is facilitated by medical officers, qualified clinical associates, and/or psychiatrists, depending on the programme [19]. Based on the consensus of the panel, this training role should be expanded to include other members of multidisciplinary team, such as psychologists and social workers. This strategy would provide clinical associates with an understanding of the types of interventions offered by different members of the multidisciplinary team, and strengthen their ability to work within multidisciplinary teams and collaborate with multidisciplinary colleagues after graduation [46, 47]. Professional groups other than doctors and nurses would likely have limited (if any) knowledge of the clinical associate cadre and a process of educating them on the role of cadre would also be required before involving them in training [15]. Consensus was reached on the importance of all of the suggested cadres being involved at the postgraduate level except medical officers and occupational therapists. Medical officers not reaching consensus is an indication that they may not be equipped to provide mental health training to clinical associates at a postgraduate level given that the psychiatry training for most of them would have been limited to their undergraduate medical degrees.

The Delphi panel reached consensus on just three work settings to provide mental health services for those with only an undergraduate degree. Unsurprisingly, this included district hospitals, as this cadre was originally developed to address health workforce shortages at the district level and their undergraduate clinical training takes place mostly at district hospitals [15, 17]. However, both CHCs and PHC clinics reached a higher degree of consensus than district hospitals, suggesting that clinical associates should have a bigger role to play at this level than they do currently. Monareng et al. [54] found that less than ten per cent of clinical associates in their study were currently practicing at PHC clinics in the public sector. The panel consensus was that it would be appropriate for clinical associates with only undergraduate mental health training and those with a postgraduate mental health qualification to provide mental health services at the PHC level (presumably with different roles). The use of this cadre at the PHC level may help address some of the issues related to integration of mental health into PHC services, such as the lack of trained staff [4, 55]. The use of clinical associates with postgraduate mental health training in GP practices may be an acknowledgement of the need to strengthen primary care mental health services in the private sector as well. The panel suggested that clinical associates with postgraduate training in mental health could have an important role to play up to the level of secondary hospitals but not beyond that. This view reflects their envisaged role at lower levels of care, where cases may be less complex and there are proportionally fewer specialist mental health professionals compared to tertiary and specialised psychiatric hospitals. The introduction of clinical associates into mental health roles in the different work settings in SA may encounter potential challenges similar to those described for mental health physician associates in the UK including role definition, integration into multidisciplinary teams, acceptance by other professionals, and lack of effective supervision [56].

The mental health tasks that the Delphi panel reached consensus on for clinical associates with only undergraduate training in mental health aligned closely with the current clinical associates’ scope of practice [16]. Taking a mental health history, performing a mental health examination and a mini-mental state examination, doing a physical examination, and providing mental health counselling all form part of their scope of practice [16]. In addition, four tasks (screening for common mental disorders, home visits to mental health patients, mental health promotion in communities, and mental health promotion in schools) that could be broadly classified as mental health promotion activities reached consensus. Promoting health at both an individual and community level is recognised as an entrustable professional activity for clinical associates [17, 57]. Health promotion competencies are emphasised for physician associates working in mental health in the UK [46, 47]. The involvement of clinical associates in mental health promotion activities in communities. as well as a role in school mental health, has been suggested by those involved in undergraduate clinical associate training programmes [27]. A role for them in screening has also been suggested, though this was limited to screening for drug and alcohol abuse and screening for mental health issues in patients with chronic medical conditions [27].

The panel saw a broad role for clinical associates with a postgraduate mental health qualification. The only task that did not reach consensus was completing the required reports for patients admitted for 72-hour observation. The lack of consensus on this item may be due to clinical associates not currently being recognised as mental health practitioners in terms of the Mental Health Care Act, 2002 which would be a requirement for completion of the 72-hour observation documentation [58]. Prescribing psychotropic medication was probably the most contentious item on the list of tasks provided, as most psychoactive medicines are registered as Schedule 5 in South Africa and clinical associates may only prescribe up to Schedule 4 - and even then, it would need to be done under the supervision of a medical practitioner [16, 59]. The panel appeared to become more supportive of the idea of clinical associates prescribing through the Delphi rounds. It has been suggested by Moodley et al. [34] that the limitation on prescribing Schedule 5 medication could be eased with appropriate training, such as a prescribing course for psychiatric medication. A prescribing course should ideally form part of the postgraduate qualification in mental health for clinical associates. A few of the other tasks on the list the panel felt were appropriate for clinical associates with a postgraduate qualification to perform, such as restraining or sedating an aggressive patient, may be considered to currently fall outside of the current scope of practice of clinical associates [16]. These Regulations [16] may need to be revisited if postgraduate training in mental health does become a reality. There would also need to be consideration given to whether it would be appropriate for a clinical associate with a postgraduate qualification in mental health to be supervised by a medical officer who is likely to only have had undergraduate training in mental health, and is likely to be the case at district and primary care level.

### Limitations

There is no definitive guidance on the ideal size of a Delphi panel, with panels of between 10 and 100 participants generally being utilised [60]. While the size of our panel was at the lower end of this range, with 26 participants in the first round dropping to 23 participants by the end of the third round, we maintained our initial target of a minimum of 10 psychiatrists and 10 family physicians throughout. The panel had a few more psychiatrists than family physicians, which may potentially have influenced the results [60]. There is also no definitive agreement on how to define consensus in a Delphi panel. We used the proportion within a range (unrestricted) approach, with 70% of participants scoring 1,2,3 or 7,8,9 regarded as consensus, though only a limited number of items reached consensus at the lower end of the range in our study [40]. It is conceivable that the results would have been different had a different definition of consensus been used. The decision on what proportion threshold to use for the proportion within a range (unrestricted) approach is also somewhat arbitrary, as higher proportions of 75% for example, have been used [40]. We opted for 70% based on a recent Delphi panel by the World Health Organization related to COVID-19 [61]. We used the DSM-5 [36] disorder categories in our study rather than individual disorders. As it was possible that participants would rate individual disorders differently within a category, we asked participants to rate the overall category according to disorder they would rate the highest within the category. This decision was made to standardise responses.

## Conclusions

The Delphi panel’s recommendations provide a clear roadmap for enhancing mental health curricula for clinical associates. The proposed approach advocates a focused selection of disorders during undergraduate training, expanding to a broader spectrum at the postgraduate level, with a strong emphasis on practical skills. The suggested delineation of mental health service provision aligns with the tiered health system, emphasising employment at primary care and district levels for clinical associates with undergraduate training. Clinical associates who complete a postgraduate qualification in mental health in the future could perform an expanded set of mental health tasks at primary care, district and regional hospital levels, offering a more comprehensive range of mental health services.

## Data Availability

The datasets used and analysed during the current study are available from the corresponding author on reasonable request.

## Declarations

### Authors qualifications

SVM is a public health medicine specialist (MBChB, FCPHM, MMed). JW has a MPH degree and a PhD in Curriculum Development and Instructional Design. CG is a psychiatrist (MBChB, FCPsych, MMed) and holds a Doctor of Medicine degree in Psychiatry.

## Abbreviations

BCMP: Bachelor of Clinical Medical Practice
BMCP: Bachelor of Medicine in Clinical Practice
CAQ: Certificate of added qualification
CHC: Community health centre
COVID-19: Coronavirus disease 2019
DSM-5: Diagnostic and Statistical Manual of Mental Disorders, Fifth Edition
GP: General practitioner
UK: United Kingdom
USA: United States of America

## References

[1] Malakoane B, Heunis J, Chikobvu P, Kigozi N, Kruger W (2020). Public health system challenges in the Free State, South Africa: a situation appraisal to inform health system strengthening. BMC Health Serv Res.

[2] Govender K, Girdwood S, Letswalo D, Long L, Meyer-Rath G, Miot J (2021). Primary healthcare seeking behaviour of low-income patients across the public and private health sectors in South Africa. BMC Public Health.

[3] Republic of South Africa. Department of Health. National Health Act, 2003. White paper on national health insurance. Government Gazette; 2015.

[4] Sorsdahl K, Petersen I, Myers B, Zingela Z, Lund C, van der Westhuizen C (2023). A reflection of the current status of the mental healthcare system in South Africa. SSM-Mental Health.

[5] Janse van Rensburg B, Kotzé C, Moxley K, Subramaney U, Zingela Z, Seedat S (2022). Profile of the current psychiatrist workforce in South Africa: establishing a baseline for human resource planning and strategy. Health Policy Plann.

[6] Docrat S, Besada D, Cleary S, Daviaud E, Lund C (2019). Mental health system costs, resources and constraints in South Africa: a national survey. Health Policy Plann.

[7] South Africa. Department of Health (2013). National Mental Health Policy Framework and Strategic Plan 2013–2020.

[8] Herman AA, Stein DJ, Seedat S, Heeringa SG, Moomal H, Williams DR. The South African stress and health (SASH) study: 12-month and lifetime prevalence of common mental disorders. South Afr Med J. 2009;99(5). PMC3191537 19588796

[9] Hunt X, Breet E, Stein D, Tomlinson M. The COVID-19 pandemic, hunger, and depressed mood among South africans. National Income Dynamics (NIDS)-Coronavirus Rapid Mobile Survey (CRAM). Wave 2021; 5.

[10] World Health Organization (2018). Mental health atlas 2017.

[11] Mental health atlas-2017 country profiles [https://www.who.int/mental_health/evidence/atlas/profiles-2017/en/] (accessed 1 September 2019).

[12] Wishnia J, Strugnell D, Smith A, Ranchod S (2019). The supply of and need for medical specialists in South Africa.

[13] De Kock JH, Pillay BJ (2017). A situation analysis of psychiatrists in South Africa’s rural primary healthcare settings. Afr J Prim Health care Family Med.

[14] De Kock J, Pillay BJ (2016). Mental health nurses in South Africa’s public rural primary care settings: a human resource crisis. Rural Remote Health.

[15] Couper ID, Hugo JF (2014). Addressing the shortage of health professionals in South Africa through the development of a new cadre of health worker: the creation of Clinical associates. Rural Remote Health.

[16] South Africa (2016). Department of Health. Health professions Act, 1974 (act No. 56 of 1974). Regulations defining the scope of practice of clinical associates.

[17] Tshabalala Z, Smalley S, Louw M, Capati J, Cooke R (2019). Clinical associates in South Africa: optimising their contribution to the health system. South Afr Health Rev.

[18] Capati J, Crichton A, Louw M, Smalley S, Tshabalala Z, Report of the Clinical Associate National Task Team 2017 (2017). Clinical associate Training and Profession-current successes and future steps.

[19] Moodley SV, Wolvaardt J, Grobler C (2022). Enabling mental health task-sharing: a collective case study of undergraduate clinical associate training programmes in South Africa. BMC Med Educ.

[20] Jenkins R, Kiima D, Okonji M, Njenga F, Kingora J, Lock S (2010). Integration of mental health into primary care and community health working in Kenya: context, rationale, coverage and sustainability. Mental Health Family Med.

[21] Federal Democratic Republic of Ethiopia. Ministry of Health. National Mental Health Strategy 2012/13–2015/16. Addis Ababa: Ministry of Health; 2012.

[22] Ahrens J, Kokota D, Mafuta C, Konyani M, Chasweka D, Mwale O, Stewart RC, Osborn M, Chikasema B, Mcheka M (2020). Implementing an mhGAP-based training and supervision package to improve healthcare workers’ competencies and access to mental health care in Malawi. Int J Mental Health Syst.

[23] Smith JM (2019). Physician assistants in psychiatry: helping to meet America’s mental health needs: well-trained PAs can help alleviate the psychiatrist shortage and improve patient care. Curr Psychiatry.

[24] Canadian Association of Physician Assistants. CanMEDS-PA. Ottawa: Canadian Association of Physician Assistants; 2015.

[25] Chidambaram AMP, Crimlisk H, Vinjamuri I, Cooney J, Wilson J (2019). Physician associates working in mental health.

[26] Gill K, Kauser S, Khattack K, Hynes F. Physician associate: new role within mental health teams. J Mental Health Train Educ Pract 2014; 9(2).

[27] Moodley SV, Wolvaardt J, Grobler C (2022). Mental health task-sharing in South Africa - a role for clinical associates?. BMC Health Serv Res.

[28] Moodley SV, Wolvaardt J, Grobler C. Mental illness attitudes, service provision interest and further training preferences of clinical associates. South Afr Family Pract. 2024;66(1). 10.4102/safp.v66i1.5808 PMC10839205 38299522

[29] University of Witwatersrand. Postgraduate Programmes. [https://www.wits.ac.za/health/academic-programmes/postgraduate-programmes/] (accessed 2023 June 13).

[30] Abbo C, Okello E, Nakku J (2013). Effect of brief training on reliability and applicability of Global Assessment of functioning scale by Psychiatric clinical officers in Uganda. Afr Health Sci.

[31] Ngungu J, Beezhold J (2009). Mental health in Zambia-challenges and way forward. Int Psychiatry.

[32] Muula AS (2009). Case for clinical officers and medical assistants in Malawi. Croatian Med J.

[33] National Commission on Certification of Physician Assistants (2018). PAs in specialty practice: an analysis of need, growth and future.

[34] Moodley SV, Wolvaardt J, Grobler C (2023). Knowledge, confidence, and practices of clinical associates in the management of mental illness. South Afr J Psychiatry.

[35] Moodley SV, Wolvaardt J, Grobler C (2023). Strengthening a mental illness management questionnaire for clinical associates through expert validation and cognitive interviews. South Afr J Psychiatry.

[36] American Psychiatric Association (2013). Diagnostic and statistical Manual of Mental disorders.

[37] Iqbal S, Pipon-Young L (2009). Methods-the Delphi method–A guide from Susanne Iqbal and Laura Pipon-Young. Psychologist.

[38] Hasson F, Keeney S, McKenna H (2000). Research guidelines for the Delphi survey technique. J Adv Nurs.

[39] Massaroli A, Martini JG, Lino MM, Spenassato D, Massaroli R. The delphi method as a methodological framework for research in nursing. Texto Contexto-Enfermagem. 2017;26(4).

[40] Diamond IR, Grant RC, Feldman BM, Pencharz PB, Ling SC, Moore AM, Wales PW (2014). Defining consensus: a systematic review recommends methodologic criteria for reporting of Delphi studies. J Clin Epidemiol.

[41] Frambach JM, van der Vleuten CP, Durning SJ (2013). AM last page: quality criteria in qualitative and quantitative research. Acad Med.

[42] Republic of South Africa (2023). Department of Health. National Mental Health Policy Framework and Strategic Plan 2023–2030.

[43] Walters K, Raven P, Rosenthal J, Russell J, Humphrey C, Buszewicz M (2007). Teaching undergraduate psychiatry in primary care: the impact on student learning and attitudes. Med Educ.

[44] GBD Mental Disorders Collaborators (2022). Global, regional, and national burden of 12 mental disorders in 204 countries and territories, 1990–2019: a systematic analysis for the global burden of Disease Study 2019. Lancet Psychiatry.

[45] Kaminer D, Grimsrud A, Myer L, Stein DJ, Williams DR (2008). Risk for post-traumatic stress disorder associated with different forms of interpersonal violence in South Africa. Soc Sci Med.

[46] National Collaborating Centre for Mental Health (2022). The competence framework for physician associates in mental health: full listings of competencies.

[47] National Collaborating Centre for Mental Health (2022). The competence framework for physician associates in mental health: Curriculum.

[48] Lampe L, Coulston C, Walter G, Malhi G (2010). Up close and personal: medical students prefer face-to-face teaching in psychiatry. Australasian Psychiatry.

[49] Guerandel A, McCarthy N, McCarthy J, Mulligan D, Lane A, Malone K (2021). An approach to teaching psychiatry to medical students in the time of Covid-19. Ir J Psychol Med.

[50] Khoo T, Warren N, Jenkins A, Turner J (2021). Teaching medical students remotely during a pandemic–what can psychiatry offer?. Australasian Psychiatry.

[51] Nayak A (2015). Changing medical students’ attitudes to psychiatry through newer teaching techniques. Mens sana Monogr.

[52] Trullàs JC, Blay C, Sarri E, Pujol R (2022). Effectiveness of problem-based learning methodology in undergraduate medical education: a scoping review. BMC Med Educ.

[53] McParland M, Noble LM, Livingston G (2004). The effectiveness of problem-based learning compared to traditional teaching in undergraduate psychiatry. Med Educ.

[54] Monareng V, Wolvaardt J, Bac M, Webb EM (2019). Practice choices of clinical associates: policy realisation or practical reality?. South Afr Med J.

[55] Wakida EK, Talib ZM, Akena D, Okello ES, Kinengyere A, Mindra A, Obua C (2018). Barriers and facilitators to the integration of mental health services into primary health care: a systematic review. Syst Reviews.

[56] National Collaborating Centre for Mental Health (2022). The competence framework for physician associates in mental health: supporting document.

[57] University of Witwatersrand Division of Clincal Associates (2023). Undergraduate outcomes and objectives.

[58] Republic of South Africa. No. 17 of 2002: Mental Health Care Act, 2002. Government Gazette; 2002.

[59] Padayachee N, Rothberg A, Butkow N, Truter I (2020). Over-the-counter Medicine utilization by beneficiaries under medical schemes in South Africa. Drug Healthc Patient Saf.

[60] Avella JR (2016). Delphi panels: Research design, procedures, advantages, and challenges. Int J Doctoral Stud.

[61] World Health Organization (2021). A clinical case definition of post COVID-19 condition by a Delphi consensus.

